# Occurrence of immune thrombocytopenic purpura in a patient with essential thrombocythemia: How the immune system can overcome a neoplastic clone

**DOI:** 10.1002/ccr3.3121

**Published:** 2020-07-17

**Authors:** Antonio Carruale, Francesco Longu, Francesca Mura, Giovanni Caocci, Giorgio La Nasa, Claudio Fozza

**Affiliations:** ^1^ Department of Medical, Surgical and Experimental Sciences University of Sassari Sassari Italy; ^2^ Department of Medical Sciences University of Cagliari Cagliari Italy

**Keywords:** essential thrombocythemia, immune thrombocytopenic purpura

## Abstract

Our case highlights the possible coexistence of essential thrombocythemia (ET) and idiopathic thrombocytopenic purpura (ITP), two pathological entities with opposite clinical and laboratory manifestations. It also underlines how an autoimmune attack has been temporarily able to overcome a neoplastic clone.

## Graphical abstract

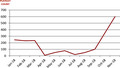

## 1 INTRODUCTION

Essential thrombocythemia (ET) and idiopathic thrombocytopenic purpura (ITP) are hematologic disorders with opposite manifestations. Our case describes the development of ITP in a patient with ET, highlighting the possible coexistence of these pathological entities and how an autoimmune attack has been temporarily able to overcome a neoplastic clone.

Essential thrombocythemia (ET) and idiopathic thrombocytopenic purpura (ITP) are two hematologic disorders showing apparently opposite clinical and therapeutic features. ET was first recognized in 1934 and in 1951 was classified as a myeloproliferative neoplasm by Damesheck.^1^ According to the World Health Organization (WHO), ET is characterized by a sustained elevation of platelet number (more than 450 × 10^9^/L) with the presence of megakaryocytic hyperplasia in the bone marrow.^2^ Driver mutations such as JAK2V617F (55%), CALR (20%‐25%), and MPL (5%) are often associated.^3^ Some patients with ET are asymptomatic, while others may experience vasomotor, thrombotic, or hemorrhagic disturbances involving cerebrovascular, coronary, and peripheral circulation.^4^ On the other side, ITP is an autoimmune hematological disorder characterized by severely decreased platelet count, in which platelet destruction is mediated by antiplatelet antibodies which may also affect marrow megakaryocytes.^5^ Patients usually suffer from cutaneous and/or mucous bleeding and much less rarely from potentially life‐threatening hemorrhages.^6^ The coexistence of ET and ITP has been reported in a single patient in the literature so far, moreover in association with thrombotic thrombocytopenic purpura.^7^

## 2 CASE REPORT

**FIGURE 1:**
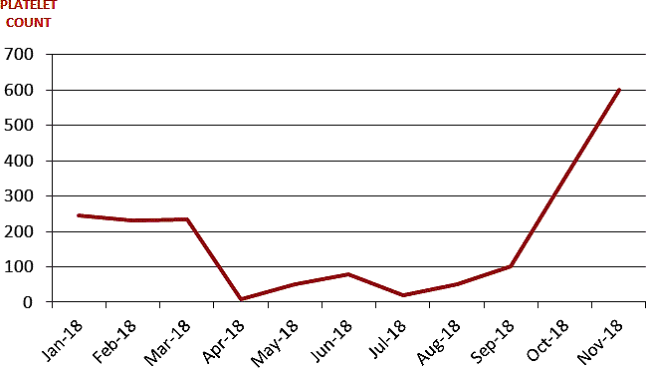
The red line represents the platelet value during the period of observation

A 95‐year‐old man was admitted to the Hematology Department of our hospital for thrombocytosis after a recent stroke, in the absence of relevant comorbidities. Hemoglobin was 12.8 g/dL, total leukocyte count 16 × 109/L, and platelet count 1.037 × 109/L. Mild splenomegaly was present. The JAK2V617 mutation with an allelic burden of 53% was identified. The patient was started on hydroxyurea and once‐daily aspirin with a good control of platelet counts. In April 2018, few weeks after confirming substantially stable blood parameters, the patient noticed the sudden appearance of multiple petechiae on his extremities. Laboratory findings were as follows: Hb 8 g/dL (MCV 113 fL), WBC 6 × 10^9^/L, platelet 7 × 10^9^/L, and reticulocytes 1.5%. No schistocytes, dacrocytes, or blasts were present on peripheral blood smear. Folate deficiency was present (<3 ng/mL) and could likely justify the condition of anemia. Serum biochemistry and coagulation were unremarkable. Hemolysis markers and screening for HIV, HCV, HBV, CMV, EBV, and other potential infective agents were negative. Physical examination was also unremarkable with substantially stable splenomegaly. Bone marrow evaluation showed a marked increase in megakaryocytes (>2/LPF) beside a shift to young, immature, less polypoid megakaryocytes and fewer mature platelet‐producing megakaryocytes, being therefore compatible with peripheral thrombocytopenia. The overall clinical and laboratory picture was interpreted as an ITP. The patient was hospitalized and treated with intravenous methylprednisolone, after stopping hydroxyurea. After a partial response obtained within few days (platelet count up to 30 × 10^9^/L), a complete response was reached only after 5 months of treatment with oral prednisone. As shown in Figure 1, after 7 months form the diagnosis of ITP, the platelet count returned above the threshold of 450 × 10^9^/L and therefore hydroxyurea and aspirin were reintroduced within the dose range applied before the diagnosis of ITP. Platelet count remained stable after one further year of treatment, thus making the hypothesis of drug toxicity very unlikely.

## 3 DISCUSSION

Essential thrombocythemia is a disease of the bone marrow hematopoietic stem cell (HSC) characterized by the acquisition of somatic mutations providing a selective advantage to mutant over normal hematopoietic precursors. Therefore, the neoplastic clone becomes capable of indefinite self‐renewal and promotes myeloid differentiation to engender a myeloproliferative phenotype.^8^ It is interesting to underline how in the present case an autoimmune attack targeting the megakaryocytic lineage was indeed able to overcome a neoplastic clone. Derangement of cellular immunity is central in the pathophysiology of adult ITP and is essentially based on a T helper 1 polarization of the overall immune response converging toward an increased platelet destruction.^9^ Besides, it is well known that megakaryocytes display characteristics consistent with increased apoptosis, which correlates with a reduction in platelet production capacity which is impaired in both the bone marrow of ITP patients and in megakaryocytes produced in vitro when treated with ITP autoantibodies.^10^ On the other side, all the other potential causes of thrombocytopenia were excluded, including bone marrow fibrosis or age‐related dysplastic or hypoplastic changes as well as infective causes or hydroxyurea toxicity, which was very unlikely due to the lack of cytopenia after a further long‐standing exposure. The present case highlights the possible coexistence of these two pathological entities involving the megakaryocytic lineage with almost opposite clinical and laboratory manifestations, although no clear pathogenetic links can be hypothesized between these two disorders thus likely pointing to a fortuitous concomitant occurrence. Moreover, it underlines how in such a battle between contrary pathogenetic pressures an autoimmune attack has been at least temporarily able to overcome a neoplastic clone.

## CONFLICT OF INTEREST

None declared.

## AUTHOR CONTRIBUTIONS

AC and CF: wrote the manuscript. All authors were involved in the diagnostic workflow and patient management and reviewed the manuscript.
